# Predictors of use of pain medications for persistent knee pain after primary Total Knee Arthroplasty: a cohort study using an institutional joint registry

**DOI:** 10.1186/ar4091

**Published:** 2012-11-16

**Authors:** Jasvinder A Singh, David G Lewallen

**Affiliations:** 1 Medicine Service and Center for Surgical Medical Acute care Research and Transitions (C-SMART), Birmingham VA Medical Center, Birmingham, AL 35294, USA; 2 Department of Medicine, School of Medicine and Division of Epidemiology, School of Public Health, University of Alabama, Birmingham, AL 35294, USA; 3 Department of Orthopedic Surgery, Mayo Clinic College of Medicine, Rochester, MN 55905 USA

## Abstract

**Introduction:**

To study the use of pain medications for persistent index knee pain and their predictors after primary Total Knee Arthroplasty (TKA).

**Methods:**

The Mayo Total Joint Registry collects patient-reported data including pain medication use on all patients who undergo TKA. We used data from patients who underwent primary TKA from 1993-2005. We examined whether gender, age (reference, ≤60 yrs), body mass index (BMI; reference, <25 kg/m^2^), comorbidities measured by Deyo-Charlson index (5-point increase), anxiety and depression predicted use of pain medications (non-steroidal anti-inflammatory drugs (NSAIDs) and opioids) 2- and 5-years after primary TKA. Multivariable logistic regression additionally adjusted for operative diagnosis, American Society of Anesthesiologists (ASA) score, implant fixation and distance from the medical center.

**Results:**

7,139 of the 10,957 eligible (65%) at 2-years and 4,234 of 7,404 eligible (57%) completed questionnaires. Significant predictors of NSAIDs use were (Odds ratio (95% confidence interval)): male gender at 2- and 5-years, 0.5 (0.4, 0.6) and 0.6 (0.5, 0.8); age >70-80 years, 0.7 (0.5, 0.9), 0.6 (0.4, 0.8); and depression, 1.4 (1.0, 1.8) and 1.7 (1.1, 2.5). BMI ≥40 was associated with NSAIDs use only at 2-years, 1.6 (1.1, 2.5). Significant predictors of opioid pain medication use at 2- and 5-years were: male gender, 0.5 (0.3, 0.9) and 0.4 (0.2, 0.8); age >70-80 years, 0.3 (0.1, 0.6), 0.3 (0.1, 0.8); and anxiety, 3.0 (1.6, 5.7) and 4.0 (1.7, 9.4).

**Conclusions:**

Female gender and younger age were associated with higher risk of use of NSAIDs and opioids after primary TKA. Depression was associated with higher NSAID use and anxiety with higher opioid pain medication use after primary TKA.

## Introduction

Primary total knee arthroplasty (TKA) is an extremely successful operation associated with significant pain relief and improvement in function and quality of life [1]. The majority of patients report significant improvement in knee pain severity after primary TKA, with only 7% of patients reporting moderate-severe index knee pain 2 years after primary TKA [2]. Studies showed a reduction in the use of oral anti-inflammatory [3] and opioid pain medications [4] after TKA. This evidence supports a significant reduction in the use of pain medications after TKA.

While most patients have significant improvement in knee pain after TKA, some patients continue using pain medications for the treatment of index knee pain. Continued use of pain medications post-operatively for the index joint is a surrogate for less beneficial outcomes after arthroplasty. Use of pain medication may be associated with significant adverse events, especially in the elderly [5-7]. Limited data exist on the factors that predict the use of pain medication after TKA. In a UK study, higher body mass index (BMI) was associated with use of anti-inflammatory drugs after TKA [3]. Higher BMI and younger age were found to be associated with the use of opioid pain medications 12 months after TKA [4]. While these studies provide insights into predictors of the use of opioid pain medication and non-steroidal anti-inflammatory drugs (NSAIDs), the analysis was not adjusted for any confounders, making the results potentially biased. Studies in other patient populations have reported that depression, anxiety and medical comorbidities may be associated with chronic use of pain medications [8,9]. In our recent analyses that were adjusted for multiple confounders, we found that female gender, depression and higher body mass index (BMI) were associated with the use of NSAIDs and opioid pain medications after total hip arthroplasty (THA) [10]. Whether this is true for patients undergoing TKA is not known.

We hypothesized that patient demographics and comorbidities will be associated with the use of pain medications after TKA. Specifically, we assessed whether female gender, younger age, higher BMI, pre-operative medical comorbidities, depression and anxiety, were associated with use of NSAIDs and opioid pain medications at 2 and 5 years after TKA. We describe the methods and results as recommended in the Strengthening of Reporting in Observational studies in Epidemiology (STROBE) statement [11].

## Materials and methods

### Setting and participants

For this study, we used the data collected prospectively in the Mayo Total Joint Registry, one of the largest US joint registries, as detailed in previous studies [12,13]. This clinical registry captures data on every patient undergoing arthroplasty (knee, hip, shoulder and others) at Mayo Clinic, Rochester, Minnesota, and follows them prospectively. Thus, the study design was an observational cohort study. Starting in 1993, patient-reported outcomes (PROs) including pain and function data reported on a validated knee questionnaire [14], were captured electronically. Patients were eligible if they had undergone primary TKA at the Mayo clinic between 1993 and 2005. We used 2- and 5-year follow-up data from these patients. The time period 1993 to 2005 was chosen because data of interest have been captured electronically since 1993, and the time frame allowed capture of follow-up data. Several previous studies have reported data on pain and function from this registry [2,15-17]. The validated knee questionnaire was administered at 2 and 5 years postoperatively by mail or in person at the clinical follow-up visit. Patients who did not return the mailed survey and failed to return for follow-up clinic visits, had the knee questionnaire administered on the telephone by dedicated Joint Registry staff. The Institutional Review Board at the Mayo Clinic, Rochester, approved the study.

### Outcomes of interest

The outcomes of interest were the use of pain medications for index TKA pain at 2- and 5-years post-primary TKA. Use of NSAIDs and opioid pain medications was assessed separately, since risk factors for their continued use after primary TKA may differ. Patients were asked the following question, 'Do you use any of the following medications for the pain in your operated knee?', and were asked to select one of the following responses: 'none', 'non-steroidal anti-inflammatory drugs (NSAIDs)', 'narcotics', or 'oral steroids'. We used none/oral steroid as the reference category and use of opioids and use of NSAIDs separately as outcomes.

### Predictors of interest

These included important patient characteristics, including age and gender (unmodifiable), and clinical factors, including BMI, medical comorbidities, depression and anxiety (modifiable). Age was categorized, as previously [2,18] into ≤ 60, 61 to 70, 71 to 80 and > 80 years. BMI was categorized, as previously [19] into ≤ 25, 25.1 to 29.9, 30 to 34.9, 35 to 39.9 and ≥ 40 kg/m^2^. We used the Deyo-Charlson score, a validated comorbidity measure [20]; this was treated as a continuous variable, and we assessed the association of a 5-point increase with outcomes. This is the most commonly used comorbidity measure consisting of a weighted scale of 19 comorbidities (including cardiac, pulmonary, renal, hepatic disease, diabetes, cancer, HIV etcetera), expressed as a summative score [21,22]. Depression and anxiety were assessed by the presence or absence before the TKA, of International Classification of Diseases, ninth revision (ICD-9) codes for depression and anxiety. ICD-9 codes were obtained from patient's entire medical record, including, but not limited to, notes from the orthopedic surgeon, anesthesiologist and other health care providers.

### Data sources

Data on the dates of the TKA, demographic details (age, gender, BMI), operative diagnosis, implant fixation, distance from the medical center and American Society of Anesthesiologists (ASA) class were obtained from the Mayo Total Joint Registry, since they are captured for every patient. ICD-9 codes for the Deyo-Charlson comorbidities, anxiety and depression were obtained the Mayo Clinic electronic databases. Median household income was calculated using the zip code data from the Mayo Total Joint Registry and the census data for median income for the respective year of surgery.

### Bias

We tried to minimize confounding bias by including factors either previously known, or suspected to be associated with use of pain medication after arthroplasty, but recognize that residual confounding is a limitation of study design. We examined for collinearity of variables in the analyses. We used generalized estimating equations (GEE) to account for correlation of observations (due to bilateral TKA in patients, performed simultaneously or sequentially) in our dataset. We anticipated non-response to be higher at 5 than 2 years, and acknowledge this as a study limitation.

### Sample size

No formal sample size calculations were done. We wanted a large enough sample to study the use of NSAIDs and opioid pain medications without having too long a study period, therefore chose all eligible patients from 1993 to 2005.

### Statistical analyses

Baseline clinical and demographic characteristics were compared using Student's *t*-test for continuous data and the chi-square test for categorical measures. Responder and non-responder characteristics were compared using logistic regression analyses. Missing data were treated as missing, and not imputed.

Multivariable adjusted logistic regression analyses were performed for NSAIDS use and opioid pain medication use at 2 and 5 years after primary TKA. For these analyses, we used a GEE approach to adjust for the correlation between observations on the same subject due to replacement of both knees. In addition to adjusting for primary predictors of interest, including age, gender, BMI, medical comorbidities, anxiety and depression, all logistic regression analyses were also adjusted for the following potential confounders: (1) operative diagnosis; (2) cemented, uncemented, or hybrid implant fixation; (3) ASA class [23] I or II vs. III or IV; (4) median household income level (≤ $35K, > $35 to $45K, > $45K) determined using the zip code and the median household income for geographical area using the census data for the respective year of the survey, as performed previously [17]; and (4) distance from the medical center of <100, 100 to 500 and > 500 miles/overseas. Distance from the medical center was included because the Mayo Clinic provides TKA to local residents, and also a serves as a referral center for patients traveling from afar; these patients may have different disease severity and expectations, which may impact pain outcomes and the use of pain medication.

We calculated Spearman's coefficient for correlation between key variables suspected to have collinearity, as follows: ASA class and Deyo-Charlson score, 0.22; ASA class and age, 0.19; age and Deyo-Charlson score, 0.14. Thus, all clinically important variables were included in the multivariable-adjusted analyses. All variables included in models were those based on clinical importance, rather than statistical significance. Goodness of fit was calculated by quasi likelihood function, a low value indicating a better fit. Odds ratios and 95% confidence intervals are presented. A *P*-value < 0.05 and 95% confidence intervals not including unity was taken to indicate statistical significance. Since the results of univariate and multivariable-adjusted analyses were similar, we present both types of analyses, but focus mainly on multivariable-adjusted estimates.

## Results

### Characteristics of the study population and the non-responders

**Figure 1:**
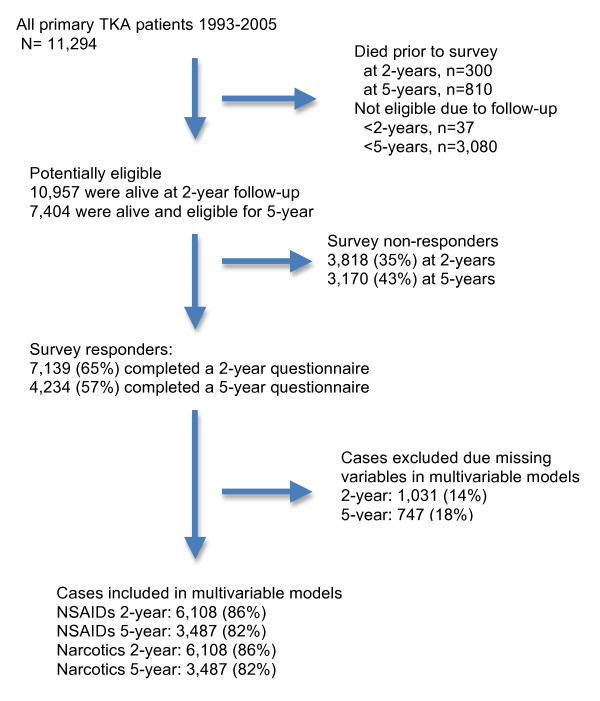
**Study cohort flow chart**. TKA, total knee arthroplasty; NSAID, non-steroidal anti-inflammatory drug.

Of the 11,294 patients who underwent primary TKA, 10,957 were alive at 2-year follow-up; of these, 7,139 (65%) completed the 2-year questionnaire (Figure 1); 7,404 of the patients were alive and eligible for 5-year follow-up, and 4,234 (57%) completed the 5-year questionnaire. Primary TKA patients responding to the questionnaire had a mean age of 68 years; 87% were overweight or obese (BMI ≥ 25), and 55 to 56% were female (Table 1). Osteoarthritis was the commonest underlying diagnosis and majority of the implants were cemented. Non-responders were more likely to be younger, have diagnosis other than osteoarthritis, have higher ASA class (III or IV), higher Deyo-Charlson comorbidity index score, and live > 500 miles from the Mayo Clinic (Additional file 1).

**Table 1:** Characteristics of patients with primary total knee arthroplasty

	Two-year follow-up(*n *= 7,139)	Five-year follow-up(*n *= 4,234)
Mean age, yrs (± SD)	68 ± 10	68 ± 10
Men/women (%)	44/56	45/55
Bilateral (%)	20	23
Age groups (%)		
≤ 60 yrs	18	18
> 60 to 70 yrs	35	37
> 70 to 80 yrs	38	38
> 80 yrs	8	7
Body mass index, kg/m^2 ^(%)		
< 25.0	13	13
25.0 to 29.9	35	36
30.0 to 34.9	29	43
35.0 to 39.9	14	7
≥ 40.0	9	7
ASA score (%)		
Class I to II	58	58
Class III to IV	42	41
Cemented (%)		
Yes	98.0	99.5
Hybrid	2.0	0.5
Underlying diagnoses (%)		
Rheumatoid arthritis/ other^a ^Inflammatory arthritis conditions	4	4
Osteoarthritis	94	93
Other	2	3

ASA, American Society of Anesthesiologists. ^a^Other includes avascular necrosis, fracture, neoplasms, Paget's disease, septic arthritis, etcetera.

### Prevalence of use of pain medications after TKA

Use of NSAIDs for index TKA pain was reported by 8.5% of patients (610/7139) at 2-year and 9% (381/4234) at 5-year follow-up. Use of opioid medication for index TKA pain was reported by 1.4% of patients (103/7139) at 2 years and 1.4% (61/4234) at 5 years.

### Predictors of use of NSAIDs 2 and 5 years after primary TKA

Univariate associations are shown in Table 2. In multivariable-adjusted analyses, at 2 years after primary TKA, significantly lower odds of NSAIDs use was noted in men with odds of 0.5, compared to women (Table 3). Compared to patients 60 years and younger, those aged 71 to 80 and > 80 years had significantly lower odds of NSAID use at 2 years after primary TKA (Table 3). Compared to patients with BMI < 25 kg/m^2^, those with BMI ≥ 40 had significantly higher odds of NSAID use 2 years after primary TKA. Presence of depression increased the odds of NSAID use by 1.4, compared to those without depression. The Deyo-Charlson comorbidity index and anxiety were not significantly associated with NSAID use.

**Table 2:** Univariate predictors of use of non-steroidal anti-inflammatory drugs after primary total knee arthroplasty

	Univariate analysis, 2-year follow-up			Univariate analysis, 5-year follow-up		
	
	Odds ratio	(95% CI)	*P*-value	Odds ratio	(95% CI)	*P*-value
Male gender (reference female)	0.51	(0.42, 0.63)	< 0.001^a^	0.66	(0.52, 0.85)	0.001^a^
Age (reference ≤ 60 yrs)						
> 60 to 70 yrs	0.78	(0.62, 0.99)	0.04^a^	0.59	(0.41, 0.80)	0.001^a^
> 70 to 80 yrs	0.60	(0.47, 0.76)	< 0.001^a^	0.54	(0.39, 0.73)	< 0.001^a^
> 80 yrs	0.52	(0.35, 0.77)	0.001^a^	0.95	(0.61, 1.49)	0.84
Body mass index (reference < 25 kg/m^2^)						
25.0 to 29.9	1.15	(0.85, 1.70)	0.30	1.13	(0.77, 1.63)	0.54
30.0 to 34.9	1.17	(0.84, 1.62)	0.35	1.02	(0.72, 1.74)	0.92
35.0 to 39.9	1.67	(1.17, 2.38)	0.005^a^	0.85	(0.54, 1.36)	0.50
≥ 40.0	2.32	(1.61, 3.35)	< 0.001^a^	1.50	(0.91, 2.48)	0.11
Deyo-Charlson index (5-point change)	1.06	(0.83, 1.36)	0.62	0.88	(0.65, 1.19)	0.40
Depression (reference no depression)	1.93	(1.51, 2.47)	< 0.001^a^	1.85	(1.32, 2.60)	< 0.001^a^
Anxiety (reference no anxiety)	1.58	(1.14, 2.19)	0.006^a^	1.13	(0.69, 1.85)	0.62

^a^Significant odds ratios and *P*-values; CI, confidence interval.

**Table 3:** Multivariable-adjusted^* ^predictors of use of non-steroidal anti-inflammatory drugs after primary total knee arthroplasty

	Multivariable-adjusted^* ^**analysis, 2-year follow-up**			Multivariable-adjusted^* ^**analysis, 5-year follow-up**		
	
	Odds ratio	(95% CI)	*P*-value	Odds ratio	(95% CI)	*P*-value
Male gender (reference female)	0.52	(0.41, 0.64)	< 0.001^a^	0.64	(0.48, 0.85)	0.002^a^
Age (reference ≤ 60 yrs)						
> 60 to 70 yrs	0.92	(0.70, 1.20)	0.53	0.57	(0.41, 0.80)	0.001^a^
> 70 to 80 yrs	0.72	(0.54, 0.95)	0.02^a^	0.56	(0.39, 0.79)	0.001^a^
> 80 yrs	0.52	(0.33, 0.82)	0.005^a^	1.00	(0.61, 1.64)	0.99
Body mass index (reference < 25 kg/m^2^)						
25.0 to 29.9	1.20	(0.85, 1.70)	0.30	1.32	(0.87, 2.00)	0.20
30.0 to 34.9	1.08	(0.76, 1.55)	0.66	1.12	(0.72, 1.74)	0.63
35.0 to 39.9	1.44	(0.98, 2.13)	0.07	0.84	(0.50, 1.41)	0.50
≥ 40.0	1.64	(1.08, 2.48)	0.02^a^	1.22	(0.68, 2.20)	0.50
Deyo-Charlson index (5-point change)	1.06	(0.80, 1.41)	0.68	0.93	(0.67, 1.31)	0.69
Depression (reference no depression)	1.39	(1.04, 1.85)	0.03^a^	1.68	(1.12, 2.51)	0.01^a^
Anxiety (reference no anxiety)	1.11	(0.77, 1.61)	0.56	0.85	(0.49, 1.47)	0.56

*Adjusted for American Society of Anesthesiologists (ASA) score, distance from the medical center, income, operative diagnosis and implant fixation in addition to all the variables in the table. ^a^Significant odds ratios and *P*-values; CI, confidence interval. Goodness of fit statistic calculated as quasi likelihood ratio with lower value indicating a better fit: 2 years, 3461.7; 5 years, 2098.4.

At 5 years after primary TKA, male gender and ages 61 to 70 and 71 to 80 years (compared to age 60 and lower) were associated with significantly lower odds of NSAID use, while depression was associated with significantly higher odds of NSAID use (Table 3). BMI, Deyo-Charlson comorbidity index and anxiety were not significantly associated with greater NSAID use. Sensitivity analyses that additionally adjusted these models for preoperative pain and functional limitation led to no change in the significance, and minimal change in the strength of association of these variables (gender, age, depression) at 2 and 5 years.

### Predictors of use of opioid pain medications 2 and 5 years after primary TKA

Univariate associations are shown in Table 4. In multivariable-adjusted analyses, at 2 years, men had odds of 0.5 of opioid pain medication use, and patients aged 71 to 80 years had odds of 0.3 (compared to those age 60 years or lower) for using opioid pain medication (Table 5). Anxiety was associated with 3.1 times odds of use of opioid pain medications 2 years after primary TKA. At 5 years after primary TKA, male gender and age 71 to 80 years were associated with lower risk, and anxiety was associated with increased risk of the use of the opioid pain medication.

**Table 4:** Univariate predictors of use of opioid pain medication after primary total knee arthroplasty

	Univariate analysis, 2-year follow-up			**Univariate analysis**, **5-year follow-up**		
	
	Odds ratio	(95% CI)	*P*-value	Odds ratio	(95% CI)	*P*-value
Male gender (reference female)	0.51	(0.42, 0.63)	< 0.001^a^	0.66	(0.52, 0.85)	0.001^a^
Age (reference ≤ 60 yrs)						
> 60 to 70 yrs	0.78	(0.35, 0.77)	0.001^a^	0.59	(0.43, 0.80)	0.001^a^
> 70 to 80 yrs	0.60	(0.47, 0.76)	< 0.001^a^	0.54	(0.39, 0.73)	< 0.001^a^
> 80 yrs	0.52	(0.33, 0.82)	0.04^a^	0.95	(0.61, 1.49)	0.84
Body mass index (reference < 25 kg/m^2^)						
2.0 to 29.9	1.15	(0.84, 1.59)	0.37	1.13	(0.77, 1.63)	0.54
30.0 to 34.9	1.17	(0.84, 1.62)	0.35	1.02	(0.69, 1.51)	0.92
35.0 to 39.9	1.67	(1.17, 2.38)	0.005^a^	0.85	(0.54, 1.36)	0.50
≥ 40.0	2.32	(1.61, 3.35)	< 0.001^a^	1.50	(0.91, 2.48)	0.11
Deyo-Charlson index (5-point change)	1.06	(0.83, 1.36)	0.62	0.88	(0.65, 1.19)	0.40
Depression (reference no depression)	1.93	(1.51, 2.47)	<0.001^a^	1.85	(1.32, 2.60)	<0.001^a^
Anxiety (reference no anxiety)	1.58	(1.14, 2.19)	0.006^a^	1.13	(0.69, 1.85)	0.62

^a^Significant odds ratios and *P*-values; CI, confidence interval.

**Table 5:** Multivariable-adjusted^* ^predictors of use of opioid pain medications after primary total knee arthroplasty

	Multivariable-adjusted^* ^analysis, 2-year follow-up			Multivariable-adjusted^* ^analysis, 5-year follow-up		
	
	Odds ratio	(95% CI)	*P*-value	Odds ratio	(95% CI)	*P*-value
Male gender (reference female)	0.54	(0.32, 0.92)	0.02^a^	0.39	(0.18, 0.84)	0.02^a^
Age (reference ≤ 60 yrs)						
> 60 to 70 yrs	0.66	(0.36, 1.20)	0.17	0.80	(0.38, 1.70)	0.57
> 70 to 80 yrs	0.27	(0.13, 0.57)	0.001^a^	0.34	(0.14, 0.83)	0.02^a^
> 80 yrs	0.48	(0.19, 1.22)	0.12	0.39	(0.10, 1.57)	0.19
Body mass index (reference < 25 kg/m^2^)						
25.0 to 29.9	1.00	(0.46, 2.17)	1.00	0.65	(0.24, 1.74)	0.39
30.0 to 34.9	1.10	(0.50, 2.44)	0.81	0.88	(0.31, 2.47)	0.80
35.0 to 39.9	1.25	(0.49, 3.22)	0.64	0.86	(0.29, 2.58)	0.79
≥ 40.0	0.54	(0.19, 1.59)	0.27	0.40	(0.10, 1.64)	0.20
Deyo-Charlson index (5-point change)	1.28	(0.70, 2.33)	0.43	1.03	(0.48, 2.21)	0.93
Depression (reference no depression)	1.05	(0.55, 2.00)	0.88	1.91	(0.83, 4.41)	0.13
Anxiety (reference no anxiety)	3.05	(1.62, 5.72)	0.001^a^	3.98	(1.68, 9.43)	0.002^a^

*Adjusted for American Society of Anesthesiologists (ASA) score, distance from the medical center, income, operative diagnosis and implant fixation in addition to all the variables in the table; ^a^Significant odds ratios and *P*-values; CI, confidence interval. Goodness of fit statistic calculated as quasi likelihood ratio with lower value indicating a better fit: 2 years, 824.3; 5 years, 519.1.

## Discussion

In this study, we found that younger age and depression were associated with higher odds of use of NSAIDs, and male gender with lower odds of use of NSAIDs for the treatment of index TKA pain, 2 and 5 years after primary TKA. BMI ≥ 40 was associated with higher NSAID use 2 years after primary TKA. Female gender, younger age and anxiety were associated with higher odds of use of opioid pain medication for the treatment of index TKA pain at both 2 and 5 years after primary TKA.

Our study has many novel findings that add to the current knowledge. The prevalence of use of pain medication at 2-year (8.5% NSAIDs and 1.4% opioids), or 5-year (9% NSAIDs and 1.4% opioids) follow-up after primary TKA in our study is similar to that reported previously in a cohort of primary THA patients from our clinical registry at the same time-points (12 to 13% NSAIDs or 2 to 3% opioids) [10]. Loss to follow-up and non-response bias may explain this discrepancy in use of pain medication after THA vs. TKA, since poorer pain outcomes are expected after TKA than after THA, based on other clinical outcomes studies of pain. The proportions are lower, as expected, than the previously reported overall 14% prevalent use of opioids 1 year after TKA [4] and 49% for use of any NSAID 2 years after TKA [3]. The use of pain medication in both these studies represented the overall use of NSAIDs or opioids compared to use of these medications for persistent index arthroplasty pain. Our study outcome captures patients with inadequate relief of index arthroplasty pain leading to persistent use of either NSAIDs or opioid pain medications. This represents a clinically important outcome for patients undergoing arthroplasty. Studies are needed to examine use of these medications at long-term follow-up after TKA.

Preoperative depression predicted use of NSAIDs for index TKA pain at 2 and 5 years after primary TKA. In an unadjusted analysis, one previous study reported that female gender and younger age were associated with higher use of opioid pain medication in TKA [4]. Our study confirms this finding in a multivariable-adjusted analysis, and extends this observation to 2 and 5 years after primary TKA. Depression predicts pain at 1 year [24] and pain and function at 2-year [25] and 5-year follow-up after primary TKA [26]. To our knowledge, there are no published studies that have examined whether depression is associated with use of pain medication 2 to 5 years after primary TKA. We speculated that depression might be associated with use of NSAIDs and opioids, but our study found that depression predicted NSAIDs use and anxiety, but not depression, predicted opioid use after primary TKA. These analyses were adjusted for multiple demographic and clinical factors suggesting that this is an independent association. This finding suggests that screening for depression and anxiety prior to surgery may help identify patients at-risk for poorer pain outcomes. Further studies need to examine if preoperative optimization of depression and anxiety can reduce the use of pain medications after primary TKA.

The association of male gender with lower likelihood of use of NSAIDs and opioid pain medication merits some discussion. Lower odds of use of NSAIDs and opioids in men vs. women with primary TKA is similar to lower analgesic use reported for men compared to women in national cohorts of patients in the US and Sweden [27,28]; our study extends this finding to populations undergoing primary TKA.

The association of older age, compared to patients 60 years and younger, with lower odds of use of NSAIDs and opioids is interesting. Other studies have reported lower use of pain medication or use of lower doses and/or greater benefit with the same doses in older individuals compared to younger controls [29-34]. This may be related to higher perceived risk of associated adverse events and contraindications, higher pain thresholds and/or patient preference for use or non-medical interventions in the elderly compared to younger patients.

These findings must be interpreted in conjunction with our previously published findings of pain and function outcomes in this cohort of patients. Higher Deyo-Charlson index, female gender, higher BMI and older age were associated with worse functional limitation [17], while female gender, higher comorbidity and younger age, but not BMI, were associated with worse pain outcomes [35] after primary TKA. In the current study, we found that female gender and younger age were associated with higher odds of use of NSAIDs and opioid pain medications. The differences in factors associated with each of the three outcomes, namely the use of pain medication, moderate-severe pain and moderate-severe functional limitation, should not be surprising. Conceptually, these domains have some overlap, but these not the same or simply surrogates for each other.

Our study has several strengths. We used multivariable analyses to assess predictors of the use of NSAIDs and opioid pain medication after primary TKA in a large sample of patients from a prospective US clinical joint registry. Most previous studies were limited to 1- to 2-year follow-up, did not ask about use of medication specifically for knee pain, and did not control for important covariates that can impact the use of pain medication (including depression and anxiety). The use of pain medication after primary TKA is under-studied in the arthroplasty literature.

Our study has several limitations. Non-response and referral bias may limit generalizability to general populations. The response rate of 65% at 2 years and 56% at 5 years, is similar to the average response rate of 60% in large surveys of this size [36]. Despite controlling for several important confounders and covariates, residual confounding is possible. Currently there is no national US joint registry, and therefore an analysis of data collected over a considerable time period from a large volume medical center (such as ours) is the next best approach. We used the diagnostic codes for depression and anxiety from medical records, and these are known to be under-coded. A similar limitation applies to other diagnoses in the Deyo-Charlson index. Such misclassification bias would have made our estimates more conservative compared to true associations. The actual associations may have been even stronger, had all patients been screened for depression using validated instruments (Center for Epidemiological Studies Depression (CES-D) scale or Beck's Depression Inventory), or an examination by a psychologist. Despite a large sample size, few patients reported use of opioid medication for the index primary TKA pain, with approximately 10 to 15 patients per variable available for analysis in the multivariable-adjusted model. These results must not be over-interpreted and need to be confirmed in future studies. Non-responders to the survey may be more likely to have poorer outcomes and a higher prevalence of use of pain medication, therefore, the actual use of these medications may be higher than that reported here. Our analyses did not account for use of preoperative pain medication, which might influence postoperative use.

## Conclusions

In conclusion, using prospectively collected data from a large US joint registry, we found that female gender and younger age were associated with higher odds of use of NSAIDs and opioid pain medication for the treatment of residual pain in the index TKA, 2 and 5 years after primary TKA. Depression was associated with higher risk of NSAID use and anxiety with higher risk of use of opioid pain medication after primary TKA. Future studies should examine whether pre- and postoperative optimization of treatment of depression and anxiety can reduce the use of pain medication after primary TKA.

## Competing interests

Dr. Singh has received investigator-initiated research grants from Takeda and Savient; consultant fees from URL pharmaceuticals, Takeda, Ardea, Savient, Allergan and Novartis, and is a member of the executive of OMERACT, an organization that develops outcome measures in rheumatology, and receives arms-length funding from 36 companies. Dr Singh is also a member of the American College of Rheumatology's Guidelines Subcommittee of the Quality of Care Committee and Veterans Affairs Rheumatology Field Advisory Committee. Dr Lewallen has received royalties/speaker fees from Zimmer, has been a paid consultant and owns stock in Pipeline Biomedical, and his institution has received research funds from DePuy, Stryker, Biomet and Zimmer.

## Abbreviations

ASA: American Society of Anesthesiologists; BMI: body mass index; GEE: generalized estimating equation; ICD-9: International Classification of Diseases, ninth revision; NSAID: non-steroidal anti-inflammatory drug; PRO: patient-reported outcome; STROBE: Strengthening of Reporting in Observational studies in Epidemiology; THA: total hip arthroplasty; TKA: total knee arthroplasty.

## Authors' contributions

JAS was responsible for study concept and design, modification of study design, review and interpretation of analyses, drafting of the manuscript and made revisions to the manuscript. DGL provided modifications of study design, review and interpretation of analyses and made revisions to the manuscript. All authors read and approved the final manuscript.

## Supplementary Material

Additional file 1 **Non-responder characteristics**. This file shows the characteristics of non-responders from the primary total knee arthroplasty (TKA) cohort at 2 and 5 years. Click here for file
