# Supplementary material for: Predictors of use of pain medications for persistent knee pain after primary Total Knee Arthroplasty: a cohort study using an institutional joint registry
Source: Arthritis Res Ther. 2012 Nov 16;14(6):R248. doi: 10.1186/ar4091 (PMC3674590; doi:10.1186/ar4091)
Supplement: Additional file 1 — Non-responder characteristics. This file shows the characteristics of non-responders from the primary total knee arthroplasty (TKA) cohort at 2 and 5 years. [file ar4091-S1.DOCX]

**Additional Files**

**Additional file 1. Non-responder characteristics**

|  | **2-yr primary TKA** | | **5-yr primary TKA** | |
| --- | --- | --- | --- | --- |
|  | **Events for non-responders**  **(3818/**  **10957)** | **OR**  **(95 %)) CI)** | **Events for non-responders (3170/7404)** | **OR**  **(95 %)) CI)** |
| **Gender** |  |  |  |  |
| Women | 2184/6161 (35.4%) |  | 1860/4191 (44.4%) |  |
| Men | 1634/4796 (34.1%) | 0.94  (0.86,1.03) | 1310/3213 (40.8%) | **0.86 ‡ (0.78,0.96)** |
| **Age groups n (%)** |  |  |  |  |
| ≤60 yrs | 841/2154 (39%) |  | 728/1473 (49.4%) |  |
| >60-70 yrs | 1273/3804 (33.5%) | **0.79 ^‡^**  **(0.69,0.89)** | 1065/2641 (40.3%) | **0.69 ^‡^ (0.60,0.80)** |
| >70-80 yrs | 1387/4121 (33.7%) | **0.79 ^‡^ (0.70,0.89)** | 1142/2759 (41.4%) | **0.72 ^‡^ (0.63,0.83)** |
| >80 yrs | 317/878 (36.1%) | 0.88 (0.74,1.05) | 235/531 (44.3%) | 0.81 (0.65,1.01) |
| **BMI Categorized** |  |  |  |  |
| ≤24.9 | 514/1474 (34.9%) |  | 452/1018 (44.4%) |  |
| 25-29.9 | 1287/3766 (34.2%) | 0.97 (0.84,1.11) | 1061/2586 (41%) | 0.87 (0.74,1.02) |
| 30-39.9 | 1644/4712  (34.9%) | 1.00 (0.87,1.15) | 1346/3169 (42.5%) | 0.92 (0.79,1.08) |
| ≥40 | 355/960 (37%) | 1.10 (0.91,1.32) | 299/602 (49.7%) | 1.24 (0.99,1.55) |
| **ASA** |  |  |  |  |
| 1-2 | 2021/6136 (32.9%) |  | 1771/4238 (41.8%) |  |
| 3-4 | 1772/4778 (37.1%) | **1.20 ^‡^ (1.10,1.31)** | 1388/3129 (44.4%) | **1.11 * (1.00,1.23)** |
| **Deyo-Charlson index (5 point increase)** |  | **1.30 ^‡^ (1.17,1.44)** |  | 1.07 (0.93,1.22) |
|  |  |  |  |  |
| **Income** |  |  |  |  |
| >$45K | 1035/3099 (33.4%) |  | 720/1665 (43.2%) |  |
| ≤$35K | 699/2098 (33.3%) | 1.00 (0.87,1.14) | 736/1841 (40%) | 0.87 (0.75,1.02) |
| >$35K-$45K | 1347/4044 (33.3%) | 1.00 (0.89,1.11) | 1058/2541 (41.6%) | 0.94 (0.82,1.07) |
| **Distance** |  |  |  |  |
| 0-100 miles | 1785/5454 (32.7%) |  | 1443/3523 (41%) |  |
| >100-500 miles | 1435/4166 (34.4%) | 1.08 (0.98,1.19) | 1218/2871 (42.4%) | 1.06 (0.95,1.19) |
| >500 miles or  Non-US | 476/1017 (46.8%) | **1.81 ^‡^ (1.55,2.11)** | 382/709 (53.9%) | **1.68 ^‡^ (1.40,2.03)** |
| **Underlying Diagnoses** |  |  |  |  |
| Inflammatory Arthritis | 172/428 (40.2%) |  | 155/344 (45.1%) |  |
| Osteoarthritis | 3480/10190(34.2%) | **0.77 * (0.62,0.97)** | 2872/6794 (42.3%) | 0.89 (0.70,1.14) |
| Other | 166/338 (49.1%) | **1.44 * (1.05,1.96)** | 143/266 (53.8%) | **1.42 * (1.00,2.01)** |

^*^P<0.05; ^‡^ p<0.01, ^†^p<0.001

All other p-values are ≥0.05, unless indicated as above
